# Improved Utilisation and Quality of Blood Culture Services Following Operational Research in a Tertiary Hospital in Ghana

**DOI:** 10.3390/tropicalmed10090270

**Published:** 2025-09-18

**Authors:** Rita Sewornu, Emily Boakye-Yiadom, Emmanuel Ativi, Precious Kwablah Kwadzokpui, Bismark Senahey, Helena Owusu, Pruthu Thekkur, Ajay M. V. Kumar, Cornelius C. Dodoo

**Affiliations:** 1 Laboratory Department, Ho Teaching Hospital, Ho P.O. Box MA 374, Ghana; ebyiadom@uhas.edu.gh (E.B.-Y.); ativiemmanuel@gmail.com (E.A.); kwadzokpuiprecious@gmail.com (P.K.K.); bismarkelly@gmail.com (B.S.); 2 Department of Microbiology and Immunology, School of Medicine, University of Health and Allied Sciences, Ho P.O. Box PMB 31, Ghana; 3 Pharmacy Department, Family Health, Korle-Bu Teaching Hospital, Accra P.O. Box KB 77, Ghana; h.owusu@kbth.gov.gh; 4 Centre for Operational Research, International Union Against Tuberculosis and Lung Disease (The Union), 2 Rue Jean Lantier, 75001 Paris, France; pruthu.tk@theunion.org (P.T.); akumar@theunion.org (A.M.V.K.); 5 Centre for Operational Research, The Union South-East Asia Office, C6, Qutub Institutional Area, New Delhi 110016, India; 6 Department of Community Medicine, Yenepoya Medical College, Yenepoya (Deemed to be University), Mangalore 575018, India; 7 Department of Pharmaceutical Microbiology, School of Pharmacy, University of Health and Allied Sciences, Ho P.O. Box PMB 31, Ghana; cdodoo@uhas.edu.gh

**Keywords:** blood culture, operational research, SORT IT, bloodstream infections, neonatal sepsis, laboratory quality, antimicrobial resistance, antimicrobial susceptibility testing, Volta Region, Ghana

## Abstract

Operational research (OR) published in 2023 revealed low utilisation and suboptimal quality of blood culture services at a tertiary hospital in Ghana. To address these, several interventions were implemented, including sensitisation of physicians, training of laboratory staff, use of automated incubation systems, and improved availability of laboratory consumables. To assess the impact of these interventions, we conducted a follow-up study in a cross-section of inpatients (n = 1080) with suspected bloodstream infections (BSI) admitted in 2024. There were three key changes. First, there was a five-fold increase in requests for blood culture from 8% pre-OR to 40% post-OR. Culture requests were more frequent from child health department (63%) and intensive care units (53%) compared to surgery department (15%). Second, there was a reduction in delays: time from hospital admission to culture request was reduced from 2 days to 1 day, and the laboratory turn-around time was reduced from 7 days to 5 days. Third, there was a marginal improvement (*p* = 0.692) in the quality of blood cultures: diagnostic yield improved from 7% to 10%, and the contamination rate was reduced from 16% to 14%. Achieving universal culture utilisation among eligible patients and lowering contamination rates will require a detailed gap analysis and targeted interventions.

## 1. Introduction

Bloodstream infections (BSI) are serious infections associated with high morbidity, permanent disability, and mortality, requiring hospital admission and emergency care [1,2]. The management of BSI is complicated by the increasing trend in antimicrobial resistance (AMR) among the pathogens causing them. An estimated 47–50 million cases and 11 million deaths from BSI occur annually worldwide, and two-fifths of these occur in children under 5 years [1].

The burden of AMR in hospitalised patients with BSI is globally high, with the greatest impact in Africa [3,4]. This is critically evident in Ghana, where a recent systematic review confirmed a high prevalence of resistance observed among various bacterial species with key pathogens like *Acinetobacter* spp., exhibiting resistance to ceftriaxone of >80%. Furthermore, studies specific to BSI in Ghana have shown a rise in the prevalence of multi-drug resistant (MDR) bacteria [5,6,7]. The global action plan on AMR recognises the inappropriate use of antimicrobials in BSI as one of the drivers of AMR [8]. Therefore, identifying the causative organism in BSI and understanding its resistance patterns are essential for adapting antimicrobial therapy [9]. Additionally, blood culture and drug susceptibility testing (CDST) provide essential data for developing local antibiograms, which are critical for guiding effective empirical therapy and for surveillance of regional bacterial resistance trends [10]. It is also a critical component of the facility-level antimicrobial stewardship programme. This is especially important in resource-constrained settings like sub-Saharan Africa, where the high prevalence of BSI is often accompanied by weak diagnostics for clinical management [11]. Even though laboratory diagnostics for BSI are shifting towards using rapid diagnostic tests, including molecular tests, traditional blood cultures, and CDST remain an integral part of the diagnostic bouquet and provide opportunities for diagnostic stewardship [12].

For effective management of BSI, blood samples for cultures must be drawn prior to the initiation of empirical therapy, which can then be modified based on the CDST results [13]. However, compliance with these established protocols for the diagnosis and treatment of BSI is not strictly adhered to. Errors in suspecting BSI often lead to a delay in initiating the request for blood culture, which, in turn, can lead to a delay in diagnosis and initiation of effective treatment. The utilisation of blood culture services varies across countries and health facilities, even in resource-rich countries [14]. Also, the turnaround time (TAT) for blood cultures in laboratories needs to be fast enough to have a favourable effect on management [15]. Furthermore, high contamination rates and prolonged TAT not only delay effective treatment and worsen outcomes but also frequently lead to the unnecessary use of broad-spectrum antimicrobials, driving antimicrobial resistance and imposing significant additional costs on both patients and the healthcare system [7].

The Ho Teaching Hospital (HTH) is a tertiary health facility in Ghana that receives referrals from primary and secondary health facilities for the management of specialised and complicated cases. An operational research (OR) study at the HTH published by Boakye-Yiadom et al. in August 2023 revealed four key findings: (i) a low proportion of patients with suspected BSI had blood cultures requested (8%); (ii) a delay in requesting blood culture (median of 2 days from admission); (iii) high contamination rates in blood cultures (16%); and (iv) long laboratory turnaround times (median of seven days) [16]. The antimicrobial resistance patterns were not reported in this study due to inadequate numbers of bacteria isolated. Non-compliance with Clinical and Laboratory Standards Institute (CLSI) guidelines was observed in the selection of antimicrobials for testing. This included testing the wrong antimicrobials and omitting recommended ones [16]. Hospital exit outcomes were also not reported due to challenges in retrieving the data from the electronic health records (EHR).

Following this study, some interventions were implemented to improve the utilisation and quality of blood culture services for diagnosing BSI. These included (i) sensitisation and training of the treating physicians and nurses such that they develop a high index of suspicion for BSI and use the correct techniques for collecting blood samples; and (ii) the use of automated incubation systems and improving the availability of laboratory consumables for blood CDST. In addition, the laboratory information management system and the EHR were strengthened to better capture key variables such as antimicrobial resistance data and hospital exit outcomes.

To assess the impact of these interventions, we conducted a follow-up operational research study to assess if there has been any change in the utilisation and quality of blood culture services among inpatients with suspected BSI at the HTH in 2024. Our specific objectives were (i) to assess the number (proportion) who had a blood culture requested and performed (an indicator of utilisation); (ii) to assess the number (proportion) of specimens that were culture-positive, culture-negative, and culture-contaminated (an indicator of quality); (iii) to describe the common pathogens identified and their antimicrobial resistance patterns; (iv) to assess median duration between (a) the date of hospital admission and the date of specimen receipt at the laboratory and (b) the date of specimen receipt and the date of reporting of results (turnaround time); and (v) to describe the hospital exit outcomes and associated factors.

## 2. Materials and Methods

### 2.1. Study Design

This was a cross-sectional study with a retrospective uncontrolled before-and-after approach using secondary data from routine laboratory records and patient EHR between January and December 2024.

### 2.2. General Setting

Ghana is a tropical country situated on the west coast of Africa and has a population of 33 million [17]. The average life expectancy at birth is 64 years, and the World Bank has classified Ghana as a lower-middle-income country [17].

### 2.3. Specific Setting

The HTH, affiliated with the University of Health and Allied Sciences (UHAS), is a 306-bed capacity health facility offering tertiary healthcare services to the inhabitants of the Volta and Oti regions of Ghana and neighbouring Togo. The medical directorate comprises departments of internal medicine, surgery, obstetrics and gynaecology, child health, public health, and diagnostics and rehabilitation, an accident and emergency unit, and both adult and neonatal intensive care units. In 2024, the outpatient attendance was 212,129, and admissions were 10,936 (Source: Annual Performance Review Report, HTH, Ghana, 2024).

Laboratory services are centralised and span haematology, biochemistry, histopathology, serology, microbiology, and blood banking. Additionally, the laboratory serves as the public health reference laboratory for tuberculosis and HIV diagnosis and is part of the network of laboratories for COVID-19 testing services in the country. It is also a sentinel site for AMR surveillance in the country.

Health information, including patient, laboratory, and pharmacy records, is managed electronically—mostly with the Lightwave Health Information Management System software (LHIMS), which feeds into the District Health Information Management System (DHIMS2) of the Ghana Health Service.

While approximately 90% of the patients are covered by the National Health Insurance Scheme (NHIS), it does not fully cover the costs of all the diagnostic tests, including blood CDST services.

#### 2.3.1. Blood Culture Processing Workflow in HTH

Blood culture requests are made by the attending physician via the patient’s EHR for clinically indicated cases. Physicians or nurses obtain aerobic blood culture bottles from the bacteriology unit for inpatients; anaerobic cultures were not part of the standard protocol for this study. Blood samples were drawn from peripheral veins, usually on the forearm for both adults and children. In accordance with the site’s protocol at the time of the study, skin antisepsis was performed using a 70% ethanol solution, allowing it to dry completely. Blood was directly drawn into a syringe—1–3 mL for neonates and 8–10 mL for adults, which are volumes deemed appropriate per standard guidelines—and aseptically transferred into the aerobic culture bottle. Upon receipt in the bacteriology laboratory, all samples underwent a visual inspection to verify the adequacy of the sample volume, in addition to checking labelling completeness. Specimens were then logged into blood culture reception registers (electronic and paper-based) and assigned a unique laboratory accession number.

#### 2.3.2. Laboratory Processing of Blood Cultures

Samples received at the blood culture bench are once again checked to confirm that the information on the request form, sample label, and specimen receipt register match. The culture bottle is then scanned, placed into an automated incubator (BD Bactec™, Becton, Dickinson and Company, Dun Laoghaire, Ireland), and incubated aerobically at 35–37 °C. The incubator scans the bottles every 10 min to detect any bacterial growth. Any bottle in which growth is detected is flagged red with a corresponding alarm. After five days of incubation in the automated incubator, if there is no growth, the equipment flags green, and the culture bottle is taken out and recorded as no bacterial growth (negative). This five-day protocol is consistent with the manufacturer’s guidelines for the detection of common bacterial and fungal pathogens and aligns with the objectives of this study. The laboratory scientist on duty then brings that bottle out, disinfects the cover with 70% ethanol, and using a sterile needle draws out 0.5 mL of the fluid. This is Gram stained and sub-cultured on appropriate media (one each on blood agar, chocolate agar in a candle-extinction jar, and MacConkey agar) and incubated aerobically at 35–37 °C. Upon growth of a bacterial pathogen, these are subsequently processed by biochemical means for phenotypic identification. Even though it is recommended to test paired samples, most of the blood samples received at the laboratory are solitary samples.

Gram-positive pathogens recovered generally include *Staphylococcus aureus* and *Streptococcus* spp. Gram-negative pathogens recovered include *Escherichia coli*, *Klebsiella* spp., *Pseudomonas aeruginosa*, and *Acinetobacter* spp. Fungal pathogens are occasionally isolated and are often either *Candida* spp. or *Aspergillus* spp. Likely contaminants recovered generally include Coagulase-negative *Staphylococcus*, *Corynebacterium* spp., and *Bacillus* spp. The classification of an isolate as a potential contaminant was based on a combination of its identity as common skin flora (Coagulase-negative staphylococci [CoNS], *Corynebacterium species*, *Bacillus species*, *Micrococcus species*, or *Propionibacterium*) and a lack of clinical evidence of infection supporting its role as a pathogen; for example, the attending physician did not initiate targeted antimicrobial therapy.

Antimicrobial susceptibility testing is performed via disc diffusion as per CLSI guidelines [18]. Gram-positive organisms are tested against penicillin, cefoxitin, erythromycin, tetracycline, ciprofloxacin, gentamicin, clindamycin, azithromycin, and co-trimoxazole. Gram-negative bacteria are tested against ceftazidime, ceftriaxone, cefepime, meropenem, tobramycin, piperacillin-tazobactam, amikacin, and ciprofloxacin. The zone of inhibition in both cases is read using the INGCO Digital Caliper (www.ingoco.com, China). The quality control of antibiotic discs and testing procedures was ensured through routine use of reference strains *Escherichia coli* ATCC 25922 and *Staphylococcus aureus* ATCC 29213.

### 2.4. Study Population

We included all inpatients at the HTH from 1st January to 31st December 2024 with clinical diagnoses of suspected BSI, including sepsis, septicaemia, septic shock, pneumonia, neonatal sepsis, catheter-related BSI, puerperal sepsis, septic abortion, urosepsis, endocarditis, meningitis, intra-cerebral abscess, pyelonephritis, enteric or typhoid fever, septic arthritis, peritonitis, cholecystitis, cholangitis, biliary tract infection, typhoid/enteric fever, intra-abdominal abscess, pelvic abscess, fever/pyrexia of unknown origin, osteomyelitis, Ludwig’s angina, and Fournier’s gangrene.

### 2.5. Data Variables and Data Collection Process

In LHIMS, we searched and extracted data for all inpatients admitted during the study period. Additionally, we extracted data for all inpatients for whom blood cultures were requested and processed during the study period. We then merged the laboratory records for patients for whom blood cultures were performed using the patient hospital ID number.

Variables extracted were the hospital ID, age, gender, dates of admission and discharge, ward of admission, clinical diagnosis, whether CDST was requested (yes/no), and hospital exit outcomes. Variables for laboratory data extracted included the date of specimen receipt by the laboratory, the result of blood culture (positive, negative, contaminated), whether a final report was issued (yes/no), and the date of the final report issue. For those with positive cultures, information on bacterial isolates identified and antimicrobial susceptibility results were extracted. These entries were subsequently anonymised, assigned unique study identification numbers, and exported for analysis.

### 2.6. Data Analysis

Data were extracted from the LHIMS in Microsoft Excel format (Microsoft corporation™) and exported to Stata (version 16.0, Copyright 1985–2019, StataCorp LLC, College Station, TX, USA) for analysis. We summarised the baseline demographic and clinical data of patients using frequency and proportions for categorical variables. For the continuous variables, we used mean and standard deviation or median and interquartile range, as appropriate.

The entire cascade of care was depicted in the form of a flowchart and included the following outcome indicators: (i) proportion of patients with suspected BSI for whom cultures were requested (indicator of utilisation); (ii) proportion of specimens processed at the laboratory and among them, the culture-positive proportion, the culture-negative proportion, and the contaminated proportion (indicator of quality); and (iii) frequencies and proportions of bacteria identified. Median (IQR) durations between (a) hospital admission and culture specimen receipt and (b) culture specimen receipt and the sharing of results were calculated. Hospital exit outcomes were categorised as favourable (recovered or discharged) or unfavourable (died, lost to follow-up, referred for further care, absconded, discharged against medical advice).

To assess associations of demographic and clinical variables with the outcome indicators, we used chi-square test for comparing proportions. Multivariable analysis using modified Poisson regression with a robust variance estimator was conducted to assess the independent association of demographic and clinical characteristics of patients with (i) request for blood CDST and (ii) unfavourable hospital exit outcomes. Collinearity between the variables was assessed using variance inflation factor (VIF). Variables with a VIF of >10 were excluded from the multivariable analysis. Prevalence ratios (PR) or risk ratios (RR), as appropriate, with a 95% confidence interval (CI), were calculated as measures of association. A *p*-value < 0.05 was considered statistically significant.

## 3. Results

### 3.1. Demographic, Clinical Characteristics, and Factors Associated with Blood Culture Request

The demographic and clinical characteristics of the study population are shown in Table 1. There were a total of 1080 inpatients with suspected BSI during the study period. Of them, 524 (49%) were males and 249 (23%) were neonates. Nearly 40% of the patients were from the department of child health followed by internal medicine (24%) and accident and emergency departments (16%).

### 3.2. Utilisation and Quality of Blood Cultures

**Figure 1:**
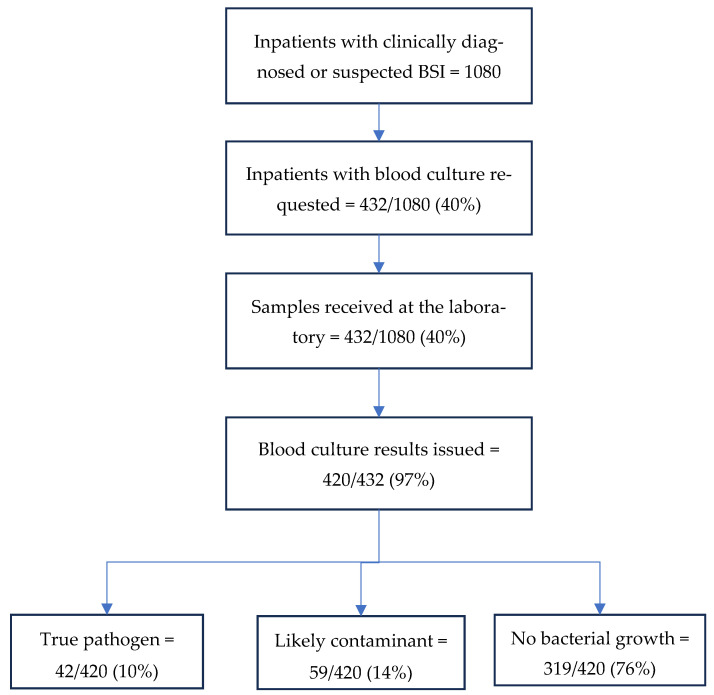
Utilisation and quality of blood cultures among inpatients with clinically suspected bloodstream infections at the Ho Teaching Hospital, Ghana, 2024.

Figure 1 depicts the indicators related to the utilisation and quality of blood cultures. Of the 1080 inpatients with clinically suspected BSI, a culture was requested by the clinicians for 432 (40%). Blood samples of all 432 patients were received by the laboratory and of them, culture results were issued for 420 (97%). Of the latter, true pathogens were isolated in 10%, likely contaminants were observed in 14%, and no growth was seen in 76% of the samples.

The blood culture processing times are shown in Table 2. The median (IQR) duration between admission and sample receipt at the laboratory was 1 (0–2) day. The median (IQR) laboratory turnaround time (time between receipt of sample at the laboratory and issuance of results) was 5 (5–6) days.

### 3.3. Organisms Isolated and Their Resistance Patterns

Of the organisms isolated, 12/42 (28%) were Gram-positive bacteria (*Staphylococcus aureus*, *Streptococcus pyogenes*), 27/42 (64%) were Gram-negative bacteria (*Pseudomonas* spp., *Acinetobacter* spp., *Achromobacter* spp., and Enterobacterales, which include *Klebsiella* spp., *Proteus mirabilis*, *Escherichia coli*, *Citrobacter* spp., *Enterobacter* spp., and *Providencia* spp.), and 3/42 (8%) were yeast-like cells. The likely contaminants were coagulase-negative *Staphylococcus* 54/59 (91.5%), *Bacillus* spp. 3/59 (5.1%), and *Micrococcus* spp. 2/59 (3.4%). Critical World Health Organization (WHO) Priority bacterial pathogens identified during the study period included third-generation cephalosporin-resistant Enterobacterales and carbapenem-resistant Enterobacterales. In addition, some isolates of methicillin-resistant *Staphylococcus aureus* (MRSA), classified as a high-priority pathogen by the WHO, were also detected. Table 3 provides a summary of the WHO priority bacterial pathogens tested and identified.

### 3.4. Factors Associated with Blood Culture Request

Table 1 depicts the factors associated with blood culture request. The likelihood of CDST being requested was significantly higher in children aged 0–11 years (62–69%), with PRs ranging from 2.5 to 2.8, indicating more than double the likelihood of CDST requests compared to patients aged 12–64 years (21–30%). Compared to pneumonia (25.3%), CDST was more likely to be requested in neonatal sepsis (60.7%), other sepsis (52.4%), and other conditions (30.0%). Patients admitted to the child health department (62.6%), intensive care units (53.3%), internal medicine (31.0%), and accident and emergency (24.4%) had significantly higher uptake of CDST compared to the surgery department (15.1%).

### 3.5. Factors Associated with Hospital Exit Outcomes

Among the 1080 inpatients with BSI, 22.5% experienced unfavourable outcomes (21.3% death, 0.6% referral for further care, 0.6% absconded). Factors associated with unfavourable outcomes are shown in Table 4. The risk of unfavourable outcomes was higher in patients aged 45 years and above compared to neonates. Patients admitted in intensive care units, accident and emergency, and internal medicine departments had significantly higher risk of unfavourable outcomes compared to those in the department of child health. Additionally, patients with “no bacterial growth” or “contaminated cultures” had significantly worse outcomes than those with confirmed bacterial infections.

## 4. Discussion

This is the first study from Ghana examining the impact of a package of interventions post-OR study on the utilisation and quality of blood culture services at a tertiary care hospital [16]. There were three key changes. First, there was a five-fold increase in request for blood culture from 8% pre-OR to 40% post-OR [16]. Second, there was a reduction in delays involved in the process: time from hospital admission to culture request was reduced from 2 days to 1 day, and the laboratory turn-around time was reduced from 7 days to 5 days. Third, while we observed a numerical increase in diagnostic yield (7% to 10%) and a decrease in contamination (16% to 14%), both metrics remain suboptimal (*p* > 0.05) and have direct, negative implications for patient care and antimicrobial stewardship. A contamination rate of 14% far exceeds the recommended target of <3% [19,20]. This high rate likely leads to the misinterpretation of contamination as bacteraemia, prompting the unnecessary use of broad-spectrum antibiotics (e.g., vancomycin) for common skin flora. This practice directly drives antimicrobial resistance, increases treatment costs, and exposes patients to potential drug-related adverse effects.

Furthermore, the persistently low diagnostic yield of 10% suggests that blood cultures are often requested for patients with a low pre-test probability of bacteremia, or that prior antibiotic use is common. This represents inefficient use of limited laboratory resources in a resource-constrained setting. Both high contamination and low yield undermine the core goals of Ghana’s National Action Plan on AMR by hindering the collection of reliable surveillance data and promoting inappropriate antimicrobial use, rather than enabling targeted, effective therapy.

Nonetheless, the observed changes were likely to be due to a package of targeted interventions implemented after the initial operational research study. These included (i) the full-scale operation of an automated blood culture incubator; (ii) addressing the procurement and supply chain gaps for culture bottles and laboratory reagents; (iii) sensitisation of the core hospital management and clinicians at various diagnostic departments, as well as a dedicated Antimicrobial Stewardship (AMS) programme under the Commonwealth Partnership Antimicrobial Stewardship (CwPAMS) programme, which raised awareness about the need to request for cultures to aid targeted treatment; and (iv) capacity-building support for the laboratory staff [21,22,23].

Under-use of blood cultures in LMICs is well-documented and largely due to limited laboratory capacity [24]. Our baseline data of 8% uptake is consistent with other Ghanaian reports of poor blood culture utilisation [13]. The post-intervention increase to 40% represents a major step towards meeting the Ghana National Standard Treatment Guidelines’ recommendation that all suspected BSIs should have cultures [25].

The uptake of cultures was significantly higher among neonates and those who were severely ill (patients admitted to intensive care units and accident and emergency departments) compared to inpatients from surgery and obstetrics and gynaecology departments. The reasons for this are unclear and need further investigation. While pre-intervention barriers such as lower clinical suspicion of BSI and hesitancy to request testing may have historically contributed to this disparity, the institution of sustained sensitisation and stewardship interventions was specifically designed to mitigate these factors by raising awareness and demonstrating utility. Therefore, the persistence of this gap post-intervention suggests that intrinsic differences in the patient case mix, where surgical and obstetrics/gynaecology patients may less frequently present with classic sepsis syndromes compared to critically ill or neonatal patients, is now a more likely explanatory factor than physician resistance. This warrants further investigation to tailor future diagnostic stewardship efforts for these specific departments. The high uptake at the child health department is similar to observations made by Rahden et al. in Gambia [26].

The overall decrease in laboratory turnaround time may be attributable to the use of automated blood culture system in the bacteriology laboratory and overall workflow improvements. Automated blood culture systems with automated alerts and continuous bottle monitoring are well known to speed up detection. Many recent studies in LMICs show that automated incubators detect >80% of positive bottles within 24 h compared to approximately 65–94% in the manual system [24]. Likewise, five days of incubation is generally sufficient in automated systems, whereas older manual protocols involved incubation for 7 days without added yield. The reduced turnaround time has important clinical implications. Faster results allow for the earlier initiation of targeted treatment and antibiotic de-escalation, where necessary [27]. The CDC guidelines note that obtaining blood cultures prior to antibiotics is key to enabling the later de-escalation of broad-spectrum therapy [20]. In a low-resource hospital such as the HTH, shaving two days off results means that clinicians can adjust treatment more quickly for better management of patients.

Despite these gains, blood culture contamination remained high at 14%, only marginally down from 16% pre-OR. This far exceeds international benchmarks such as that of the CLSI and related guidelines, which set a contamination target of <3%, with an ideal closer to 1% [20]. High contamination has several adverse effects [28]. It drives false-positive results with skin commensals, wastes laboratory and clinical resources, and can mislead therapy [28]. CDC guidance stresses that even basic steps, such as adequate skin antisepsis with 70% ethanol and allowing for time to dry, disinfecting the bottle cap, and using a good phlebotomy technique, are essential to reduce contamination rates [20]. The persistently elevated rate shows that further training and quality monitoring are needed. For instance, routine surveillance of contamination rates by ward, as recommended in infection control programmes, could identify problem areas for further interventions. One of the recommended procedures to differentiate between “true pathogens” and “likely contaminants” is the use of paired samples. This is not being practiced at the HTH owing to cost constraints and needs urgent review. Our immediate quality improvement plan, therefore, will be to implement a two-step antisepsis protocol, which involves 70% ethanol followed by povidone–iodine, and to intensify efforts within our antimicrobial stewardship programme to promote the collection of paired blood samples. This is essential to definitively differentiate true pathogens from contaminants. While paired sampling has been historically limited by cost constraints, we propose to urgently review this policy as a vital investment. Furthermore, we will introduce routine ward-specific surveillance of contamination rates to identify and target problem areas for additional interventions.

The overall blood culture positivity for true pathogens remained low (10%), though showing an increment compared to the previous study (7%). This is in line with other Ghanaian and African reports. A nationwide Ghana surveillance found only 11.2% of cultures grew pathogens, and in a large Ghana referral centre, only 9.3% of blood cultures yielded true pathogens. Low diagnostic yield suggests cultures being requested for patients with a low probability of bacteremia, exposure to antibiotics prior to culture, or limited incubation of blood cultures to a maximum of five days, which may have failed to detect some slow-growing pathogens. Prior studies in Ghana have noted that early empirical antibiotics, often given before culture request, and even misdiagnosis of other febrile illnesses, contributes to low yields. It is also possible that inadequate blood volume reduced sensitivity. Low yield has clinical implications; thus, most patients with negative cultures will be treated empirically. Without a confirmed pathogen, clinicians lack definitive guidance to narrow therapy, underscoring the need to improve diagnostic accuracy. Diagnostic stewardship is therefore vital, and blood CDST requests should be reserved for patients with true clinical sepsis and obtained before antibiotics are started to maximise both yield and the utility of the results [29]. In practical terms, our findings argue for refining the indications for blood culture, for example, using clinical prediction rule, ensuring adequate draw volume, and reviewing the protocol for duration of blood culture to include a 14-day incubation time for patients with suspected endocarditis.

The pathogen and CDST data are concerning for emerging resistance. Although numbers were small, most (five out of seven) of the *Staphylococcus aureus* isolates were methicillin-resistant (MRSA). This high prevalence is likely a direct consequence of the selective pressure exerted by the widespread empirical use of broad-spectrum antibiotics. This needs to be considered while recommending the antibiotics used for empirical therapy. Among Gram-negatives, resistance to third-generation cephalosporins was high. This parallels other Ghana findings, such as that reported in a cancer centre where 61% of Enterobacteriaceae were cefotaxime-resistant (mostly ESBLs) [30], and earlier (2010–13) data from Korle-Bu Teaching Hospital showed a year-on-year rise in cephalosporin-resistant Enterobacteriaceae among BSIs [31]. One isolate in our series was even resistant to carbapenems. While rare, carbapenem-resistant bloodstream pathogens have been reported in Ghana and are regarded as critical threats. The WHO’s 2024 priority pathogens list highlights carbapenem-resistant Gram-negatives and MRSA as top priorities [32]. These local resistance patterns underscore the danger of relying on outdated empiric regimens. In the absence of real-time culture data, clinicians may be unknowingly prescribing ineffective antibiotics, which may, in turn, be leading to poor outcomes. As shown in our study, patients with a definitive diagnosis had better outcomes compared to those with “contaminated” or “negative” results.

The broad antimicrobial resistance observed here, including high MRSA and resistance to third-generation cephalosporins, thus heightens the urgency of stewardship and justifies the emphasis in Ghana’s National Action Plan that empirical therapy should be guided by up-to-date local antibiograms.

The overall mortality rate of 21.3% observed in our study aligns with the grave prognosis associated with bloodstream infections (BSI) in similar resource-limited settings across sub-Saharan Africa. A study from a large tertiary hospital in Addis Ababa, Ethiopia, reported a stark fivefold increase in mortality among patients with a positive blood culture (50.0% vs. 9.8% for culture-negative patients) [33]. This pattern of high mortality is further exacerbated by antimicrobial resistance (AMR); the same Ethiopian study reported a devastating 100% mortality rate among all 11 patients infected with third-generation cephalosporin-resistant Enterobacteriaceae [33]. While direct causal comparisons cannot be drawn, the consistent theme across these geographically distinct African cohorts is that BSI mortality remains critically high and is profoundly influenced by AMR, which cripples the efficacy of standard empiric therapy. This collective evidence underscores the urgent need for improved diagnostic stewardship and access to effective antibiotics to mitigate this persistent threat to patient survival in our region.

This study has several strengths. First, we had a large sample and included everyone with suspected BSI during the study period, thus enabling robust analysis of data including factors associated with culture request and hospital exit outcomes. Second, we used routine hospital data which are likely to reflect ground realities. Third, we followed the STROBE (Strengthening the Reporting of Observational Studies in Epidemiology) guidelines in conducting and reporting the results of our study.

There are some limitations too. First, the overall numbers of true pathogens were small. Hence, caution is needed when interpreting the magnitude of resistance patterns observed. Second, the number of demographic and clinical factors available to assess associations with culture uptake and outcomes were limited (with some of them being collinear to each other). Third, we did not investigate the reasons for lower uptake from certain departments such as surgery and obstetrics and gynaecology. This needs further investigation using qualitative inquiry. However, the uneven uptake of blood culture across departments, reflecting real-world clinical practice, limits the comparability of AMR patterns and outcomes between these patient groups. The findings on resistance patterns are therefore most representative of the patient populations in high-uptake departments (e.g., Child Health, ICU) and may not be generalizable to all inpatients with suspected BSI. Fourth, using the interval from admission to issuance of laboratory report as a proxy for diagnostic lead time was based on the assumption that all patients had these infections at admission and did not account for possible healthcare-associated infections. Furthermore, the high proportion of solitary blood culture samples received by the laboratory represents a key limitation. The inability to obtain paired samples from separate venipuncture sites impedes our ability to reliably differentiate true bloodstream infection from contamination, particularly for common skin commensals like coagulase-negative staphylococci. This likely affects the precision of our reported contamination rate and the clinical interpretation of some isolates.

Despite these limitations, our study has many implications and provides the basis for several recommendations. First, we need to investigate the reasons for the gap in the uptake of blood culture and act based on these findings. Moving from 40% to 100% will not be easy and will require a combination of approaches, which include (i) the targeted engagement of departments that are not optimally involved; (ii) a review of the cost structure of CDST services and collaboration with the Ministry of Health to offer it to patients free of charge; and (iii) the augmentation of traditional diagnostics such as CDST with rapid molecular and advanced identification systems such as Vitek—this is likely to reduce the turnaround times [34]. Second, there is an urgent need to revisit the clinical criteria of suspected BSI to ensure culture services are offered and prioritised to the needy patients—this will improve the diagnostic yield. Third, the practice of testing paired blood samples should be promoted. This will help in differentiating contaminants from true pathogens and in the institution of targeted therapy. Finally, information on the resistance patterns of bacteria should be used to periodically update the empirical therapy regimens.

## 5. Conclusions

In conclusion, we found that a package of interventions based on the findings of an operational research study led to a major improvements in utilisation (five-fold increase in uptake of blood culture) and modest improvements in the quality of blood culture services—namely, a reduction in laboratory turnaround times and a marginal reduction in contamination rates—in a tertiary hospital in Ghana. Several recommendations have been made to achieve universal coverage of all patients with bloodstream infections with blood culture services.

## Figures and Tables

**Table 1:** Demographic and clinical characteristics associated with blood culture request among inpatients with suspected bloodstream infections at the Ho Teaching Hospital, Ghana, 2024.

	Total N(%) ^		CDST Request N(%) *		PR (95% CI)	aPR ^β^ (95% CI)	*p*-Value
Total	1080	(100)	432	(40.0)			
**Age**							
Neonate (0–28 days)	249	(23.1)	155	(62.3)	2.5 (1.9–3.3)		
29 days to <1 year	39	(3.6)	24	(61.5)	2.5 (1.8–3.5)		
1–4 years	77	(7.1)	48	(62.3)	2.5 (1.9–3.4)		
5–11 years	54	(5.0)	37	(68.5)	2.8 (2.0–3.7)		
12–24 years	114	(10.6)	29	(25.4)	1.0 (0.7–1.5)		
25–44 years	172	(15.9)	37	(21.5)	0.9 (0.6–1.3)		
45–64 years	173	(16.0)	52	(30.1)	1.2 (0.9–1.7)		
≥65 years	202	(18.7)	50	(24.8)	1		
**Sex**							
Male	524	(48.5)	207	(39.5)	1	1	
Female	556	(51.5)	225	(40.5)	1.0 (0.9–1.2)	1.0 (0.9–1.1)	0.843
Condition ***							
Neonatal sepsis	242	(22.4)	147	(60.7)	2.4 (2.0–2.9)	1.4 (1.1–1.7)	**0.003**
Pneumonia	368	(34.1)	93	(25.3)	1	1	
Other sepsis ^1^	227	(21.0)	119	(52.4)	2.1 (1.7–2.6)	2.0 (1.6–2.4)	**<0.001**
Other conditions ^2^	243	(22.5)	73	(30.0)	1.2 (0.9–1.5)	1.4 (1.1–1.7)	**0.011**
**Department**							
Intensive Care Unit	30	(2.8)	16	(53.3)	3.5 (2.1–5.8)	3.5 (2.2–5.7)	**<0.001**
Accident and Emergency	168	(15.6)	41	(24.4)	1.6 (1.0–2.6)	1.8 (1.1–2.8)	**0.013**
Child Health	422	(39.1)	264	(62.6)	4.1 (2.8–6.1)	4.2 (2.9–6.2)	**<0.001**
Internal Medicine	261	(24.2)	81	(31.0)	2.1 (1.4–3.1)	2.4 (1.6–3.7)	**<0.001**
Obstetrics and Gynaecology	47	(4.4)	7	(14.9)	1.0 (0.5–2.1)	0.9 (0.4–2.0)	0.867
Surgery	152	(14.1)	23	(15.1)	1	1	

N = Total number of inpatients with suspected blood stream infections. ^ Column percentage. * Row percentage with category sub-total as the denominator. ^β^ Adjusted analysis using binomial regression; age variable was removed from the adjusted model as it was found to be collinear with other variables with a variance inflation factor > 10. Statistically significant associations are shown in bold. CDST = culture and drug susceptibility testing. PR = prevalence ratio. *** Only one diagnosis retrieved per patient. ^1^ Other sepsis = septic shock, severe sepsis, sepsis, septicaemia, puerperal sepsis, septic abortion, or sepsis following abortion. ^2^ Other conditions = endocarditis, pyelonephritis, typhoid fever, typhoid perforation, osteomyelitis, septic arthritis, intra-abdominal or pelvic abscesses, bacterial peritonitis, cholecystitis, spontaneous bacterial peritonitis, peritonitis, fever/pyrexia of unknown origin, Ludwig’s angina, and Fournier’s gangrene.

**Table 2:** Blood culture and drug susceptibility test processing time (in days) for inpatients with suspected blood stream infections in the Ho Teaching Hospital, Ghana during 2024.

Duration Between	n *	Median Days	(IQR)
Admission and receipt of blood sample at laboratory for CDST	394	1	(0–2)
Receipt of blood sample at laboratory and issuance of CDST report ^#^	390	5	(5–6)
Admission to issuance of final CDST report	373	6	(6–8)

* n, total number of observations with valid date for calculation of duration at each of the stages. CDST: Culture and drug susceptibility testing. IQR: Interquartile range. ^#^ Laboratory turnaround time (TAT).

**Table 3:** WHO priority pathogens isolated from blood cultures of inpatients with suspected blood stream infections at Ho Teaching Hospital, Ghana, 2024.

Organism	WHO Priority Category	Number of Isolates	Number Tested	Number Resistant
Macrolide-resistant *Streptococcus pyogenes*	Medium	2	1	0
MRSA (methicillin-resistant *Staphylococcus aureus*)	High	10	7	5
Carbapenem-resistant *Pseudomonas aeruginosa*	High	4	4	0
Carbapenem-resistant *Acinetobacter baumanii*	Critical	3	1	0
Carbapenem-resistant Enterobacterales	Critical	19	10	1
Ceftriaxone-resistant Enterobacterales	Critical	19	8	6
Ceftazidime-resistant Enterobacterales	Critical	19	8	4

**Table 4:** Factors associated with unfavourable hospital exit outcomes among inpatients with suspected bloodstream infections in the Ho Teaching Hospital, Ghana, 2024.

	Total N	Unfavourable Outcome n(%) *		RR (95% CI)	aRR ^β^ (95% CI)	*p*-Value
Total	1080	243	(22.5)			
**Age**						
Neonate (0–28 days)	249	24	(9.6)	1		
29 days to <1 year	39	2	(5.1)	0.5 (0.1–2.2)		
1–4 years	77	2	(2.6)	0.3 (0.1–1.1)		
5–11 years	54	2	(3.7)	0.4 (0.1–1.6)		
12–24 years	114	22	(19.3)	2.0 (1.2–3.4)		
25–44 years	172	42	(24.4)	2.5 (1.6–4.0)		
45–64 years	173	68	(39.3)	4.1 (2.7–6.2)		
>=65 years	202	81	(40.1)	4.1 (2.7–6.3)		
**Sex**						
Male	524	114	(21.7)	1	1	
Female	556	129	(23.2)	1.1 (0.9–1.3)	1.0 (0.8–1.2)	0.700
Condition ***						
Neonatal sepsis	242	18	(7.4)	1	1	
Pneumonia	368	94	(25.5)	3.4 (2.1–5.5)	0.7 (0.3–1.6)	0.427
Other sepsis ^1^	227	107	(47.1)	6.3 (4.0–10.1)	1.3 (0.6–2.9)	0.496
Other conditions ^2^	243	24	(9.9)	1.3 (0.7–2.4)	0.4 (0.2–0.9)	**0.024**
Department **						
Intensive Care Unit	30	29	(96.7)	15.1 (10.4–21.9)	14.3 (7.5–27.6)	**<0.001**
Accident and Emergency	168	72	(42.9)	6.7 (4.5–10.3)	7.1 (3.7–13.6)	**<0.001**
Child Health	422	27	(6.4)	1	1	
Internal Medicine	261	83	(31.8)	5.0 (3.3–7.5)	5.4 (2.8–10.3)	**<0.001**
Obstetrics and Gynaecology	47	9	(19.2)	3.0 (1.5–6.0)	2.8 (1.2–6.8)	**0.019**
Surgery	152	23	(15.1)	2.4 (1.4–4.0)	3.1 (1.5–6.4)	**0.002**
**Culture Status**						
No culture/no results	660	148	(22.4)	1.6 (0.7–3.2)	1.7 (1.0–3.2)	0.071
No bacterial growth	319	74	(23.2)	1.6 (0.8–3.5)	2.0 (1.1–3.6)	**0.024**
Contaminant	59	15	(25.4)	1.8 (0.8–4.2)	2.1 (1.1–4.0)	**0.037**
True bacterial pathogen	42	6	(14.3)	1		

* Row percentage with total as the denominator. ^β^ Adjusted analysis using Poisson regression; age variable was removed from the adjusted model as it was found to be collinear with other variables with a variance inflation factor > 10. Statistically significant associations are shown in bold. RR = risk ratio. ** Clinical department of ward first admitted. *** Only one diagnosis retrieved per patient. ^1^ Other sepsis = septic shock, severe sepsis, sepsis, septicaemia, puerperal sepsis, septic abortion, or sepsis following abortion. ^2^ Other = endocarditis, pyelonephritis, typhoid fever, typhoid perforation, osteomyelitis, septic arthritis, intra-abdominal or pelvic abscesses, bacterial peritonitis, cholecystitis, spontaneous bacterial peritonitis, peritonitis, fever/pyrexia of unknown origin, Ludwig’s angina, and Fournier’s gangrene.

## Data Availability

Requests to access these data should be sent to the corresponding author.

## References

[1] Seni J., Mwakyoma A.A., Mashuda F., Marando R., Ahmed M., DeVinney R., Pitout J.D.D., Mshana S.E. (2019). Deciphering risk factors for blood stream infections, bacteria species and antimicrobial resistance profiles among children under five years of age in North-Western Tanzania: A multicentre study in a cascade of referral health care system. BMC Pediatr..

[2] Martinez R.M., Wolk D.M., Hayden R.T., Carroll K.C., Tang Y.-W. (2016). Bloodstream Infections. Microbiol. Spectr..

[3] Cassini A., Högberg L.D., Plachouras D., Quattrocchi A., Hoxha A., Simonsen G.S., Colomb-Cotinat M., Kretzschmar M.E., Devleesschauwer B., Cecchini M. (2019). Attributable deaths and disability-adjusted life-years caused by infections with antibiotic-resistant bacteria in the EU and the European Economic Area in 2015: A population-level modelling analysis. Lancet Infect. Dis..

[4] de Kraker M.E.A. (2023). Understanding the impact of antimicrobial resistance on outcomes of bloodstream infections in low- and middle-income countries. PLoS Med..

[5] Deku J.G., Dakorah M.P., Lokpo S.Y., Orish V.N., Ussher F.A., Kpene G.E., Eshun V.A., Agyei E., Attivor W., Osei-Yeboah J. (2019). The Epidemiology of Bloodstream Infections and Antimicrobial Susceptibility Patterns: A Nine-Year Retrospective Study at St. Dominic Hospital, Akwatia, Ghana. J. Trop. Med..

[6] Obeng-Nkrumah N., Labi A.-K., Addison N.O., Labi J.E.M., Awuah-Mensah G. (2016). Trends in paediatric and adult bloodstream infections at a Ghanaian referral hospital: A retrospective study. Ann. Clin. Microbiol. Antimicrob..

[7] Zasowski E.J., Bassetti M., Blasi F., Goossens H., Rello J., Sotgiu G., Tavoschi L., Arber M.R., McCool R., Patterson J.V. (2020). A Systematic Review of the Effect of Delayed Appropriate Antibiotic Treatment on the Outcomes of Patients with Severe Bacterial Infections. Chest.

[8] World Health Organization (2023). Implementing the Global Action Plan on Antimicrobial Resistance: First Quadripartite Biennial Report.

[9] Maki G., Smith I., Paulin S., Kaljee L., Kasambara W., Mlotha J., Chuki P., Rupali P., Singh D.R., Bajracharya D.C. (2020). Feasibility study of the world health organization health care facility-based antimicrobial stewardship toolkit for low- and Middle-Income countries. Antibiotics.

[10] Krapp F., Rondon C., Amaro C., Barco-Yaipén E., Valera-Krumdieck M., Vásquez R., Briones A., Casapia M., Burgos A., López F.S. (2022). Underutilization and Quality Gaps in Blood Culture Processing in Public Hospitals of Peru. Am. J. Trop. Med. Hyg..

[11] Droz N., Hsia Y., Ellis S., Dramowski A., Sharland M., Basmaci R. (2019). Bacterial pathogens and resistance causing community acquired paediatric bloodstream infections in low- and middle-income countries: A systematic review and meta-analysis. Antimicrob. Resist. Infect. Control.

[12] Pan H.-W., Li W., Li R.-G., Li Y., Zhang Y., Sun E.-H. (2018). Simple sample preparation method for direct microbial identification and susceptibility testing from positive blood cultures. Front. Microbiol..

[13] Opintan J.A., Newman M.J. (2017). Prevalence of antimicrobial resistant pathogens from blood cultures: Results from a laboratory based nationwide surveillance in Ghana. Antimicrob. Resist. Infect. Control.

[14] Minton J., Clayton J., Sandoe J., Gann H.M., Wilcox M. (2008). Quality Improvement Report: Improving early management of bloodstream infection: A quality improvement project. BMJ Br. Med. J..

[15] Idelevich E., Seifert H., Sundqvist M., Scudeller L., Amit S., Balode A., Bilozor A., Drevinek P., Tufan Z.K., Koraqi A. (2019). Microbiological diagnostics of bloodstream infections in Europe—An ESGBIES survey. Clin. Microbiol. Infect..

[16] Boakye-Yiadom E., Najjemba R., Thekkur P., Labi A.-K., Gil-Cuesta J., Asafo-Adjei K., Mensah P., van Boetzelaer E., Jessani N.S., Orish V.N. (2023). Use and Quality of Blood Cultures for the Diagnosis of Bloodstream Infections: A Cross-Sectional Study in the Ho Teaching Hospital, Ghana, 2019–2021. Int. J. Environ. Res. Public Health.

[17] (2024). Ghana 2024 Statistical Year Overview Ghana Statistical Service. https://statsghana.gov.gh/gssmain/fileUpload/pressrelease/Ghana%20Statistical%20Service%202024%20Releases%20-%20online.pdf.

[18] Lewis J.S. (2024). Performance Standards for Antimicrobial Susceptibility Testing.

[19] Halstead D.C., Sautter R.L., Snyder J.W., Crist A.E., Nachamkin I. (2020). Reducing Blood Culture Contamination Rates: Experiences of Four Hospital Systems. Infect. Dis. Ther..

[20] Center for Disease Control and Prevention (2022). Blood Culture Contamination: An Overview for Infection Control and Antibiotic Stewardship Programs Working with the Clinical Laboratory. https://www.cap.org/laboratory-improvement/accreditation/.

[21] Fraser J., Skone-James R., Foster J., Rosado H., Brandish C., Nabiryo M. (2024). Commonwealth Partnerships for Antimicrobial Stewardship (CWPAMS) Impact Report. https://commonwealthpharmacy.org/.

[22] McErlean M., Kpokiri E., Panesar P., Cooper E.E., Jato J., Orman E., Odoi H., Hutton-Nyameaye A., Somuah S.O., Folitse I. (2025). Evaluation of a Hub-and-Spoke Model to Enhance Healthcare Professionals’ Practice of Antimicrobial Stewardship (AMS) Programmes in the Volta Region of Ghana. Antibiotics.

[23] Ashiru-Oredope D., Nabiryo M., Zengeni L., Kamere N., Makotose A., Olaoye O., Townsend W., Waddingham B., Matuluko A., Nambatya W. (2023). Tackling antimicrobial resistance: Developing and implementing antimicrobial stewardship interventions in four African commonwealth countries through a health partnership model. J. Public Health Afr..

[24] Ombelet S., Kpossou G., Kotchare C., Agbobli E., Sogbo F., Massou F., Lagrou K., Barbé B., Affolabi D., Jacobs J. (2022). Blood culture surveillance in a secondary care hospital in Benin: Epidemiology of bloodstream infection pathogens and antimicrobial resistance. BMC Infect. Dis..

[25] (2017). Standard Treatment Guidelines. www.ghndp.org.

[26] Rahden P., Barrow E., Bah H., Bittaye S.O., Nygren D., Badjan A. (2025). Bloodstream infections at a tertiary hospital in the Gambia—A one-year retrospective study. BMC Infect. Dis..

[27] Hsu P.-H., Chang R., Yin C.-H., Chen Y.-S., Chen J.-S. (2024). Association between blood culture turnaround time and clinical prognosis in emergency department patients with community acquired bloodstream infection: A retrospective study based on electronic medical records. Heliyon.

[28] Dempsey C., Skoglund E., Muldrew K.L., Garey K.W. (2019). Economic health care costs of blood culture contamination: A systematic review. Am. J. Infect. Control.

[29] Dräger S., Giehl C., Søgaard K.K., Egli A., de Roche M., Huber L.C., Osthoff M. (2022). Do we need blood culture stewardship programs? A quality control study and survey to assess the appropriateness of blood culture collection and the knowledge and attitudes among physicians in Swiss hospitals. Eur. J. Intern. Med..

[30] Donkor E.S., Muhsen K., Johnson S.A.M., Kotey F.C.N., Dayie N.T.K.D., Tetteh-Quarcoo P.B., Tette E.M.A., Osei M.-M., Egyir B., Nii-Trebi N.I. (2023). Multicenter Surveillance of Antimicrobial Resistance among Gram-Negative Bacteria Isolated from Bloodstream Infections in Ghana. Antibiotics.

[31] Labi A.-K., Obeng-Nkrumah N., Bjerrum S., Enweronu-Laryea C., Newman M.J. (2016). Neonatal bloodstream infections in a Ghanaian Tertiary Hospital: Are the current antibiotic recommendations adequate?. BMC Infect. Dis..

[32] World Health Organization (2024). WHO Bacterial Priority Pathogens List, 2024-Bacterial Pathogens of Public Health Importance to Guide Research, Development and Strategies to Prevent and Control Antimicrobial Resistance.

[33] Seboxa T., Amogne W., Abebe W., Tsegaye T., Azazh A., Hailu W., Fufa K., Grude N., Henriksen T.-H., Quach C. (2015). High mortality from blood stream infection in Addis Ababa, Ethiopia, is due to antimicrobial resistance. PLoS ONE.

[34] Barman P., Chopra S., Thukral T. (2018). Direct testing by VITEK^®^ 2: A dependable method to reduce turnaround time in Gram-negative bloodstream infections. J. Lab. Physicians.

